# *catena*-Poly[[[(diethyl­enetriamine-κ^3^ *N*,*N*′,*N*′′)­copper(II)]-μ-cyanido-κ^2^ *C*:*N*] perchlorate]

**DOI:** 10.1107/S1600536812023987

**Published:** 2012-06-13

**Authors:** Peter W. R. Corfield, Sylvia C. Yang

**Affiliations:** a Department of Chemistry, Fordham University, 441 East Fordham Road, Bronx, NY 10458, USA; b Department of Geological Sciences, The Ohio State University, Columbus, Ohio 43210, USA

## Abstract

The structure of the title salt, {[Cu(CN)(C_4_H_13_N_3_)]ClO_4_}_*n*_, is composed of copper-containing cations and perchlor­ate anions. The Cu^II^ atom shows a square-pyramidal coordination, with equatorial positions occupied by the cyanide C atom [Cu—C = 1.990 (3) Å] and the N atoms of the diethyl­enetriamine ligand (average Cu—N = 2.033 Å), while the axial position is occupied by the N atom of a *c*-glide-related cyanide group. The axial Cu—N distance of 2.340 (3) Å is longer than the equatorial distances, reflecting Jahn–Teller distortion. The Cu^II^ cations are linked by the cyanide groups into infinite chains along the *c-*axis direction. The refinement included a three-component disordered model for the perchlorate ion. Each minor site is stabilized by hydrogen bonds to N—H donors from four surrounding cations, while one O atom of the major perchlorate site forms hydrogen bonds to three of these cations.

## Related literature

There is a growing body of literature on self-assembled polymers involving copper cyanide moieties, with many examples of one- two- and three-dimensional networks, see, for example: Roof *et al.* (1968 ▶); Chestnut *et al.* (2001 ▶); Kim *et al.* (2005 ▶); Lim *et al.* (2008 ▶). Most of these structures involve Cu^I^ atoms bridged by cyanide ligands, while a smaller number are mixed-valence compounds with cyanide linkages between Cu^I^ and Cu^II^ atoms. The present structure was prepared as a model for CN^−^ binding to copper-containing proteins (Fager & Alben, 1972 ▶), and is a rare example of a Cu^II^ cyanide-bridged linear polymer, similar to the linear polymer reported by Zhan *et al.* (2007 ▶). For the CN stretching frequency, see: Alben & Farrier (1972 ▶). 
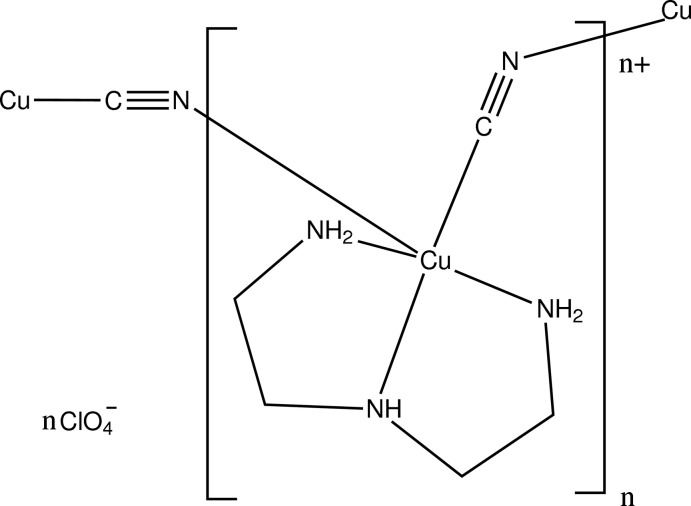


## Experimental

### 

#### Crystal data

[Cu(CN)(C_4_H_13_N_3_)]ClO_4_
*M* *_r_* = 292.18
Monoclinic, 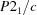

*a* = 6.7767 (8) Å
*b* = 21.5081 (16) Å
*c* = 8.3635 (12) Å
β = 118.109 (9)°
*V* = 1075.2 (2) Å^3^
*Z* = 4
Cu *K*α radiation
μ = 5.29 mm^−1^
*T* = 295 K
0.32 × 0.17 × 0.07 mm

#### Data collection

Picker four-circle diffractometer
Absorption correction: integration (Busing & Levy, 1957*a* ▶) *T*_min_ = 0.394, *T*_max_ = 0.697
3044 measured reflections
1752 independent reflections
1625 reflections with *I* > 2σ(*I*)
*R*_int_ = 0.024
6 standard reflections every 200 reflections intensity decay: none

#### Refinement

*R*[*F* ^2^ > 2σ(*F* ^2^)] = 0.031
*wR*(*F* ^2^) = 0.085
*S* = 1.09
1752 reflections
158 parameters
H-atom parameters constrained
Δρ_max_ = 0.59 e Å^−3^
Δρ_min_ = −0.35 e Å^−3^

### 

Data collection: locally modified program (Corfield, 1972 ▶); cell refinement: locally modified program (Corfield, 1972 ▶); data reduction: cell refinements and data reduction follow procedures in Corfield *et al.* (1967 ▶) and Corfield & Shore (1973 ▶); standard deviations of intensities include an ignorance factor (Busing & Levy, 1957*b* ▶) set here to 0.06; program(s) used to solve structure: local superposition program (Corfield, 1972 ▶); program(s) used to refine structure: *SHELXL97* (Sheldrick, 2008 ▶); molecular graphics: *ORTEPII* (Johnson, 1976 ▶); software used to prepare material for publication: *SHELXL97*.

## Supplementary Material

Crystal structure: contains datablock(s) I, global. DOI: 10.1107/S1600536812023987/pk2408sup1.cif

Structure factors: contains datablock(s) I. DOI: 10.1107/S1600536812023987/pk2408Isup2.hkl

Additional supplementary materials:  crystallographic information; 3D view; checkCIF report

## Figures and Tables

**Table 1:** Selected bond lengths (Å)

Cu—C8	1.990 (3)
Cu—N1	2.023 (2)
Cu—N4	2.034 (2)
Cu—N7	2.040 (3)
Cu—N8^i^	2.340 (3)
C8—N8	1.139 (4)

Symmetry code: (i) 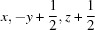.

**Table 2:** Hydrogen-bond geometry (Å, °)

*D*—H⋯*A*	*D*—H	H⋯*A*	*D*⋯*A*	*D*—H⋯*A*
N1—H1*B*⋯O3^ii^	0.90	2.38	3.215 (5)	154
N4—H4⋯O3^iii^	0.91	2.42	3.214 (5)	145
N7—H7*A*⋯O3^i^	0.90	2.23	3.092 (4)	161
N1—H1*A*⋯O4′	0.90	2.17	2.771 (17)	124
N1—H1*B*⋯O3′^ii^	0.90	2.04	2.913 (15)	164
N4—H4⋯O1′^iii^	0.91	2.30	3.139 (19)	154
N7—H7*A*⋯O3′^i^	0.90	2.14	3.040 (16)	173
N1—H1*A*⋯O1′′	0.90	2.21	3.06 (4)	156
N1—H1*B*⋯O4′′^ii^	0.90	2.51	3.21 (3)	135
N4—H4⋯O3′′^iii^	0.91	2.12	2.99 (3)	160
N7—H7*A*⋯O3′′^i^	0.90	2.45	3.24 (3)	147
N7—H7*B*⋯O4′′	0.90	2.50	3.04 (3)	119

Symmetry codes: (i) 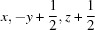; (ii) 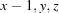; (iii) 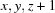.

**Fig. 1.**
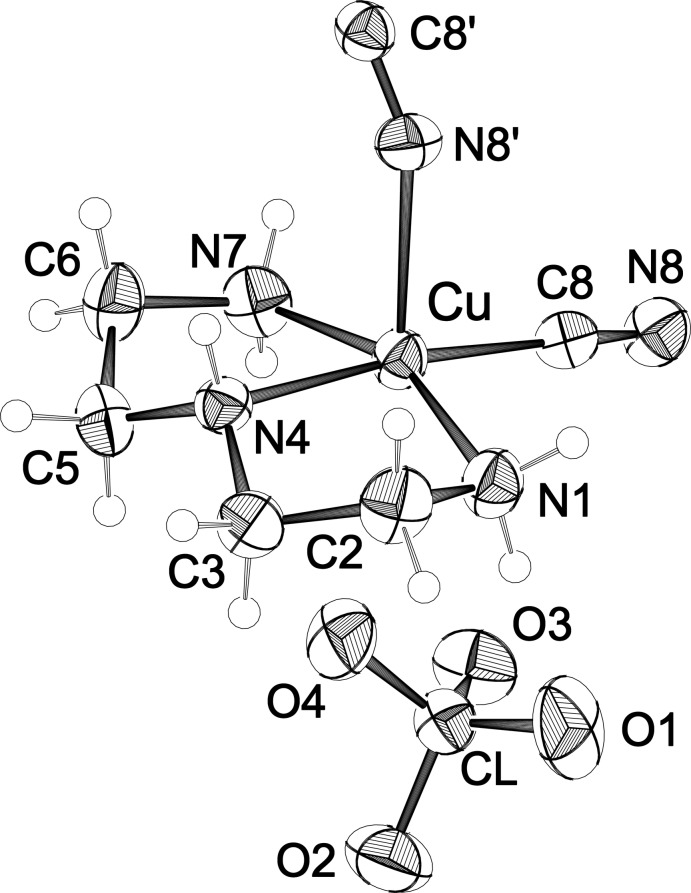
The molecular structure of the [Cu(dien)CN] cation and the major component of the perchlorate anion, with ellipsoids at the 50% level. Atoms C8' and N8' are C8 and N8 at x, 1/2-y, 1/2+z.

**Fig. 2.**
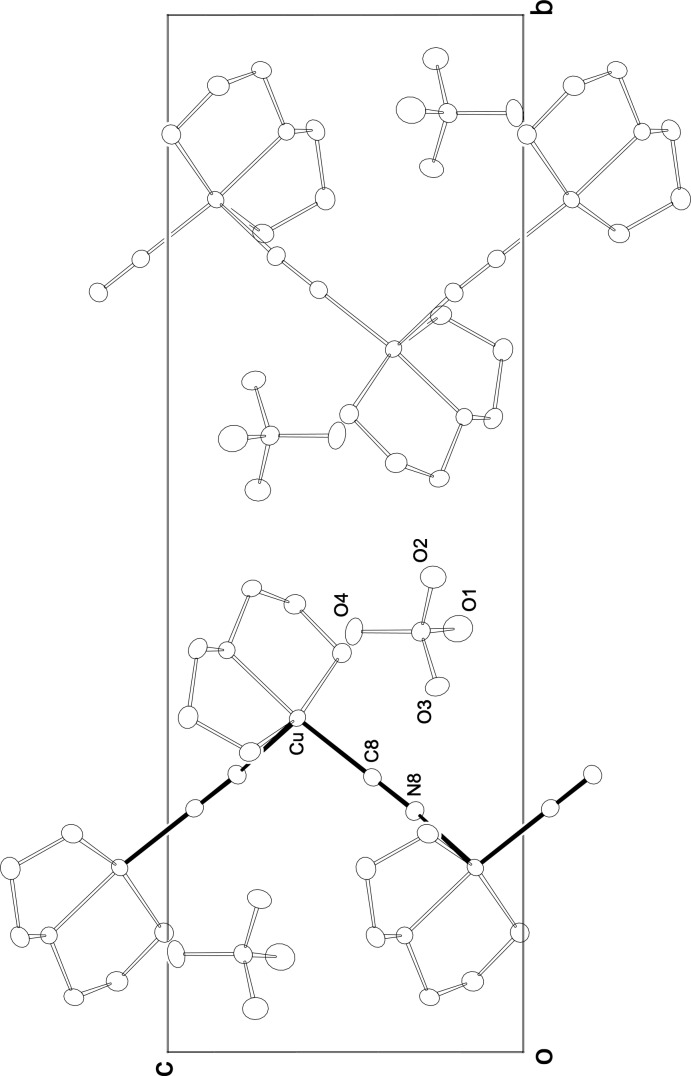
Packing of [Cu(dien)CN]ClO4, viewed down the a axis. The darkened bonds show the Cu-CN-Cu chains along the c axis. Only the major perchlorate component is shown.

**Fig. 3.**
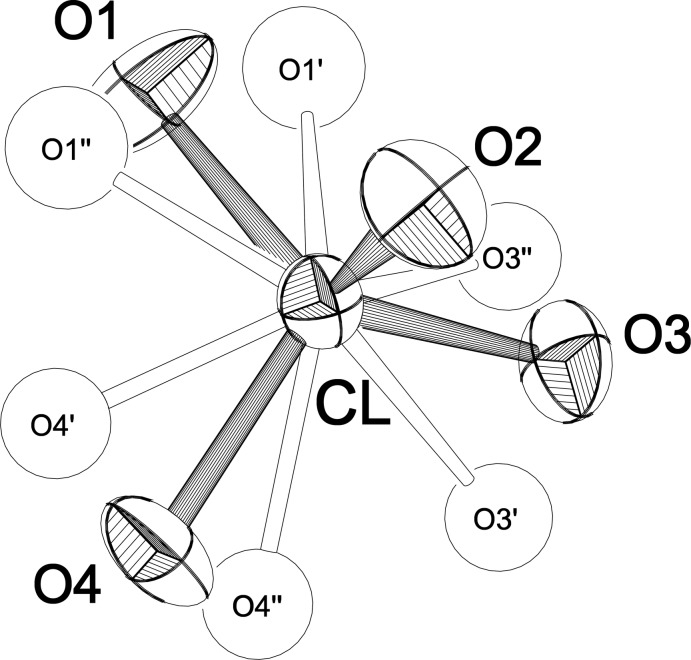
The disordered perchlorate anion, with ellipsoids drawn at the 25% probability level.

## supplementary crystallographic information

### Comment

The title compound, [Cu(dien)CN]ClO_4_, (Fig. 1), was originally prepared as a simple model for CN^-^ binding to copper-containing proteins, with the expectation that structural data would supplement information from infra-red studies on cyanide binding to the proteins. (Fager and Alben, 1972) The structure is reported now in light of current interest in cyanide-bridged copper polymers.

The crystal structure consists of cyanidodiethylenetriaminecopper(II) cations and perchlorate anions. The cyanide groups link *c*-glide related copper atoms to form infinite chains along the *c* axis, as shown in Fig. 2. The coordination of the copper atoms is square pyramidal, with the terdentate diethylenetriamine ligand and the carbon atom of the cyanide group in equatorial positions, and the nitrogen atom of a symmetry-related cyanide group in the axial position.

Atom O4 of the perchlorate group would occupy the sixth coordination site of the Cu^II^ atom if the Cu—O4 distance of 2.956 (4) Å represented a chemical bond, making the copper atom octahedrally coordinated. Perchlorate anions rarely coordinate, however, and we prefer the square pyramidal designation, in view of the long Cu—O4 distance, and the displacement of the copper atom by 0.237 (1) Å towards the axial nitrogen atom and away from the perchlorate O4 atom. Furthermore, the O4 atom has similar *U*_eq_ values to the other perchlorate oxygen atoms, and is disordered in the same way, whereas bonding to the Cu atom would be expected to localize the atom O4.

The Cu—C—N angle at the cyanide carbon atom is close to linear, at 175.9 (3)°, but the C—N—Cu angle at the bridging cyanide nitrogen atom is 146.5 (2)°, significantly different from 180°. The C—N bond length is 1.139 (4) Å, similar to the terminal bond length of 1.129 Å in K_3_Cu(CN)_4_, (Roof *et al.*, 1968).

In the diethylenetriamine ligand, the carbon atoms in each chelate ring lie on opposite sides of the corresponding CuN_2_ plane. The Cu—N1—C2—C3—N4 chelate ring has the λ conformation, with torsional angle N1—C2—C3—N4 equal to -51.9 (3) °, while the other chelate ring has the δ conformation, with the N4—C5—C6—N7 torsional angle equal to +51.9 (3) °.

Two minor alternative orientations for the perchlorate anion were refined, related to the major orientation by rotation about the Cl—O2 bond, by 34° in one direction, and 25° in the other. (Fig. 3) Each minor site is stabilized by hydrogen bonds to N—H donors from four surrounding cations, while atom O3 of the major perchlorate site forms hydrogen bonds to three of these cations.

### Experimental

The compound was prepared by addition of stoichiometric amounts of diethylenetriamine and potassium cyanide to a solution of copper(II) perchlorate. Calculated elemental analysis, based upon C_5_H_13_ClCuN_4_O_4_: C, 20.55%; H, 4.48%; N, 19.18%. Found: C, 20.60, 20.66%; H, 4.98, 4.58%; N, 19.02%. The CN stretching frequency was 2141.4 cm^-1^ (Alben and Farrier, 1972).

### Refinement

All 13 hydrogen atoms of the diethylenetriamine ligand were found unambiguously in a difference Fourier map, and were initially refined freely. In the final refinements, hydrogen atoms were constrained to idealized positions by *SHELXL97*.

The assignment of C and N atoms in the cyanide group was checked early in the analysis by carrying out a least-squares refinement with the N and C atoms of the cyanide group reversed. The weighted *R* factor increased significantly from 0.061 to 0.091. There is no evidence of disorder between the C and N atoms of the cyanide group.

Perchlorate ion disorder: Refinement of a single anisotropic perchlorate group converged successfully with wR2=0.1091 for all 1752 reflections. The thermal parameters indicated large librations about the Cl—O2 bond however, and difference Fourier maps indicated two minor alternative orientations for the perchlorate group.

After initial stringent constraints, the three orientations were refined freely, with common Cl and O2 atoms. The main orientation (O1—O4) was refined anisotropically, with an occupancy fixed at 70%. The two minor orientations (O1'-O4') and (O1''-O4'') were given occupancy factors of 18% and 12% respectively, based upon heights found in difference Fourier maps. U values for O1' and O1'', O3' and O3'', and O4' and O4'' were constrained to be equal. This model reduced wR2 significantly from 0.1091 to 0.0847, with the addition of 22 new parameters.

### Crystal data

**Table:**

[Cu(CN)(C_4_H_13_N_3_)]ClO_4_	*F*(000) = 596
*M**_r_* = 292.18	*D*_x_ = 1.806 Mg m^−^^3^*D*_m_ = 1.805 Mg m^−^^3^*D*_m_ measured by flotation in chloroform/bromoform mixtures
Monoclinic, *P*2_1_/*c*	Melting point: 471(2) K
Hall symbol: -P 2ybc	Cu *K*α radiation, λ = 1.5418 Å
*a* = 6.7767 (8) Å	Cell parameters from 25 reflections
*b* = 21.5081 (16) Å	θ = 4–52°
*c* = 8.3635 (12) Å	µ = 5.29 mm^−^^1^
β = 118.109 (9)°	*T* = 295 K
*V* = 1075.2 (2) Å^3^	Plate, dark blue
*Z* = 4	0.32 × 0.17 × 0.07 mm

### Data collection

**Table:**

Picker four-circle diffractometer	1625 reflections with *I* > 2σ(*I*)
Radiation source: sealed X-ray tube	*R*_int_ = 0.024
Oriented graphite 200 reflection monochromator	θ_max_ = 63.3°, θ_min_ = 4.1°
θ/2θ scans	*h* = −7→6
Absorption correction: integration (Busing & Levy, 1957*a*)	*k* = 0→24
*T*_min_ = 0.394, *T*_max_ = 0.697	*l* = 0→9
3044 measured reflections	6 standard reflections every 200 reflections
1752 independent reflections	intensity decay: none

### Refinement

**Table:**

Refinement on *F*^2^	Secondary atom site location: real-space vector search
Least-squares matrix: full	Hydrogen site location: difference Fourier map
*R*[*F*^2^ > 2σ(*F*^2^)] = 0.031	H-atom parameters constrained
*wR*(*F*^2^) = 0.085	*w* = 1/[σ^2^(*F*_o_^2^) + (0.010*P*)^2^ + 1.140*P*] where *P* = (*F*_o_^2^ + 2*F*_c_^2^)/3
*S* = 1.09	(Δ/σ)_max_ = 0.001
1752 reflections	Δρ_max_ = 0.59 e Å^−^^3^
158 parameters	Δρ_min_ = −0.35 e Å^−^^3^
0 restraints	Extinction correction: *SHELXL97* (Sheldrick, 2008), Fc^*^=kFc[1+0.001xFc^2^λ^3^/sin(2θ)]^-1/4^
Primary atom site location: heavy-atom method	Extinction coefficient: 0.0010 (2)

### Special details

**Table:**

Geometry. All e.s.d.'s (except the e.s.d. in the dihedral angle between two l.s. planes) are estimated using the full covariance matrix. The cell e.s.d.'s are taken into account individually in the estimation of e.s.d.'s in distances, angles and torsion angles; correlations between e.s.d.'s in cell parameters are only used when they are defined by crystal symmetry. An approximate (isotropic) treatment of cell e.s.d.'s is used for estimating e.s.d.'s involving l.s. planes.
Refinement. Refinement of *F*^2^ against all reflections. The weighted *R*-factor *wR* and goodness of fit *S* are based on *F*^2^, conventional *R*-factors *R* are based on *F*, with *F* set to zero for negative *F*^2^. The threshold expression of *F*^2^ > σ(*F*^2^) is used only for calculating *R*-factors(gt) *etc*. and is not relevant to the choice of reflections for refinement. *R*-factors based on *F*^2^ are statistically about twice as large as those based on *F*, and *R*- factors based on all data will be even larger.

### Fractional atomic coordinates and isotropic or equivalent isotropic displacement parameters (Å^2^)

**Table:**

	*x*	*y*	*z*	*U*_iso_*/*U*_eq_	Occ. (<1)
Cu	0.15547 (7)	0.322023 (18)	0.63505 (5)	0.03271 (18)	
N1	−0.0930 (4)	0.38484 (12)	0.5101 (3)	0.0426 (6)	
H1A	−0.0788	0.4034	0.4197	0.051*	
H1B	−0.2264	0.3655	0.4620	0.051*	
C2	−0.0818 (6)	0.43180 (15)	0.6424 (5)	0.0499 (8)	
H2A	−0.1495	0.4157	0.7135	0.075*	
H2B	−0.1626	0.4689	0.5799	0.075*	
C3	0.1595 (5)	0.44708 (14)	0.7639 (4)	0.0433 (7)	
H3A	0.2220	0.4689	0.6965	0.065*	
H3B	0.1743	0.4734	0.8632	0.065*	
N4	0.2770 (4)	0.38751 (10)	0.8338 (3)	0.0321 (5)	
H4	0.2433	0.3746	0.9217	0.038*	
C5	0.5219 (5)	0.38908 (15)	0.9155 (4)	0.0410 (7)	
H5A	0.5851	0.4108	1.0307	0.061*	
H5B	0.5674	0.4104	0.8360	0.061*	
C6	0.6010 (6)	0.32285 (15)	0.9427 (5)	0.0481 (8)	
H6A	0.7601	0.3214	0.9809	0.072*	
H6B	0.5750	0.3034	1.0358	0.072*	
N7	0.4756 (4)	0.28942 (12)	0.7688 (4)	0.0430 (6)	
H7A	0.4741	0.2484	0.7900	0.052*	
H7B	0.5436	0.2949	0.6997	0.052*	
C8	0.0609 (5)	0.26492 (13)	0.4243 (4)	0.0344 (6)	
N8	0.0196 (4)	0.23248 (12)	0.3050 (3)	0.0428 (6)	
Cl	0.30284 (12)	0.40553 (3)	0.28806 (10)	0.0381 (2)	
O2	0.4055 (5)	0.45800 (12)	0.2534 (4)	0.0653 (7)	
O1	0.0662 (8)	0.4083 (3)	0.1838 (8)	0.0782 (19)	0.70
O3	0.3915 (7)	0.35240 (17)	0.2420 (6)	0.0528 (10)	0.70
O4	0.3631 (8)	0.4055 (2)	0.4760 (6)	0.0593 (12)	0.70
O1'	0.109 (3)	0.3883 (9)	0.125 (3)	0.069 (5)*	0.18
O3'	0.440 (3)	0.3468 (7)	0.349 (2)	0.053 (3)*	0.18
O4'	0.238 (3)	0.4161 (8)	0.419 (2)	0.056 (4)*	0.18
O1''	0.077 (8)	0.421 (2)	0.243 (5)	0.069 (5)*	0.12
O3''	0.286 (4)	0.3518 (13)	0.183 (4)	0.053 (3)*	0.12
O4''	0.413 (5)	0.3815 (12)	0.474 (4)	0.056 (4)*	0.12

### Atomic displacement parameters (Å^2^)

**Table:**

	*U*^11^	*U*^22^	*U*^33^	*U*^12^	*U*^13^	*U*^23^
Cu	0.0355 (3)	0.0299 (3)	0.0297 (3)	0.00199 (16)	0.0128 (2)	−0.00357 (15)
N1	0.0402 (14)	0.0407 (14)	0.0389 (14)	0.0040 (11)	0.0121 (12)	−0.0018 (11)
C2	0.0497 (19)	0.0416 (17)	0.052 (2)	0.0151 (15)	0.0190 (16)	−0.0018 (15)
C3	0.0522 (19)	0.0309 (15)	0.0435 (17)	0.0043 (14)	0.0199 (15)	−0.0045 (13)
N4	0.0355 (13)	0.0314 (12)	0.0303 (12)	−0.0007 (10)	0.0163 (10)	−0.0003 (10)
C5	0.0367 (16)	0.0461 (17)	0.0388 (17)	−0.0075 (13)	0.0167 (14)	−0.0099 (14)
C6	0.0366 (17)	0.055 (2)	0.0430 (19)	0.0044 (14)	0.0104 (15)	−0.0033 (14)
N7	0.0421 (14)	0.0380 (14)	0.0494 (15)	0.0046 (11)	0.0218 (12)	−0.0046 (12)
C8	0.0376 (16)	0.0335 (15)	0.0346 (16)	0.0034 (12)	0.0191 (13)	0.0034 (13)
N8	0.0528 (16)	0.0386 (14)	0.0365 (14)	0.0052 (12)	0.0206 (12)	−0.0040 (12)
Cl	0.0391 (4)	0.0397 (4)	0.0381 (4)	−0.0016 (3)	0.0203 (3)	−0.0039 (3)
O2	0.0807 (18)	0.0531 (15)	0.0723 (17)	−0.0186 (13)	0.0446 (15)	0.0014 (13)
O1	0.030 (2)	0.077 (4)	0.095 (5)	0.003 (2)	0.003 (3)	−0.005 (4)
O3	0.068 (3)	0.0388 (19)	0.064 (3)	0.005 (2)	0.041 (3)	−0.0087 (19)
O4	0.069 (3)	0.081 (3)	0.0335 (19)	−0.017 (3)	0.029 (2)	−0.010 (2)

### Geometric parameters (Å, º)

**Table:**

Cu—C8	1.990 (3)	C5—H5B	0.9700
Cu—N1	2.023 (2)	C6—N7	1.480 (4)
Cu—N4	2.034 (2)	C6—H6A	0.9700
Cu—N7	2.040 (3)	C6—H6B	0.9700
Cu—N8^i^	2.340 (3)	N7—H7A	0.9000
N1—C2	1.474 (4)	N7—H7B	0.9000
N1—H1A	0.9000	C8—N8	1.139 (4)
N1—H1B	0.9000	N8—Cu^ii^	2.340 (3)
C2—C3	1.500 (5)	Cl—O1	1.420 (5)
C2—H2A	0.9700	Cl—O2	1.425 (2)
C2—H2B	0.9700	Cl—O3	1.426 (4)
C3—N4	1.476 (4)	Cl—O4	1.426 (4)
C3—H3A	0.9700	Cl—O1'	1.43 (2)
C3—H3B	0.9700	Cl—O3'	1.507 (15)
N4—C5	1.467 (4)	Cl—O4'	1.376 (16)
N4—H4	0.9100	Cl—O1''	1.44 (5)
C5—C6	1.501 (4)	Cl—O3''	1.42 (3)
C5—H5A	0.9700	Cl—O4''	1.46 (3)
			
C8—Cu—N1	96.45 (11)	C6—C5—H5A	110.3
C8—Cu—N4	171.38 (11)	N4—C5—H5B	110.3
N1—Cu—N4	82.98 (10)	C6—C5—H5B	110.3
C8—Cu—N7	95.09 (11)	H5A—C5—H5B	108.6
N1—Cu—N7	157.48 (11)	N7—C6—C5	108.3 (3)
N4—Cu—N7	82.73 (10)	N7—C6—H6A	110.0
C8—Cu—N8^i^	100.04 (10)	C5—C6—H6A	110.0
N1—Cu—N8^i^	100.27 (11)	N7—C6—H6B	110.0
N4—Cu—N8^i^	88.51 (9)	C5—C6—H6B	110.0
N7—Cu—N8^i^	96.66 (10)	H6A—C6—H6B	108.4
C2—N1—Cu	109.50 (19)	C6—N7—Cu	109.98 (19)
C2—N1—H1A	109.8	C6—N7—H7A	109.7
Cu—N1—H1A	109.8	Cu—N7—H7A	109.7
C2—N1—H1B	109.8	C6—N7—H7B	109.7
Cu—N1—H1B	109.8	Cu—N7—H7B	109.7
H1A—N1—H1B	108.2	H7A—N7—H7B	108.2
N1—C2—C3	108.2 (3)	N8—C8—Cu	175.9 (3)
N1—C2—H2A	110.1	C8—N8—Cu^ii^	146.5 (2)
C3—C2—H2A	110.1	O1—Cl—O2	111.1 (3)
N1—C2—H2B	110.1	O1—Cl—O3	111.4 (3)
C3—C2—H2B	110.1	O1—Cl—O4	109.3 (3)
H2A—C2—H2B	108.4	O2—Cl—O3	105.7 (2)
N4—C3—C2	106.9 (2)	O2—Cl—O4	108.1 (2)
N4—C3—H3A	110.3	O3—Cl—O4	111.1 (3)
C2—C3—H3A	110.3	O1'—Cl—O2	109.3 (8)
N4—C3—H3B	110.3	O1'—Cl—O3'	104.4 (10)
C2—C3—H3B	110.3	O1'—Cl—O4'	107.9 (11)
H3A—C3—H3B	108.6	O2—Cl—O3'	116.7 (6)
C5—N4—C3	116.4 (2)	O2—Cl—O4'	113.6 (7)
C5—N4—Cu	109.27 (17)	O3'—Cl—O4'	104.3 (9)
C3—N4—Cu	110.01 (18)	O1''—Cl—O2	108.8 (18)
C5—N4—H4	106.9	O1''—Cl—O3''	105.5 (17)
C3—N4—H4	106.9	O1''—Cl—O4''	108 (2)
Cu—N4—H4	106.9	O2—Cl—O3''	114.8 (11)
N4—C5—C6	107.1 (2)	O2—Cl—O4''	116.1 (12)
N4—C5—H5A	110.3	O3''—Cl—O4''	102.8 (15)
			
N1—C2—C3—N4	−51.9 (3)	N4—C5—C6—N7	51.9 (3)

Symmetry codes: (i) *x*, −*y*+1/2, *z*+1/2; (ii) *x*, −*y*+1/2, *z*−1/2.

### Hydrogen-bond geometry (Å, º)

**Table:**

*D*—H···*A*	*D*—H	H···*A*	*D*···*A*	*D*—H···*A*
N1—H1*B*···O3^iii^	0.90	2.38	3.215 (5)	154
N4—H4···O3^iv^	0.91	2.42	3.214 (5)	145
N7—H7*A*···O3^i^	0.90	2.23	3.092 (4)	161
N1—H1*A*···O4′	0.90	2.17	2.771 (17)	124
N1—H1*B*···O3′^iii^	0.90	2.04	2.913 (15)	164
N4—H4···O1′^iv^	0.91	2.30	3.139 (19)	154
N7—H7*A*···O3′^i^	0.90	2.14	3.040 (16)	173
N1—H1*A*···O1′′	0.90	2.21	3.06 (4)	156
N1—H1*B*···O4′′^iii^	0.90	2.51	3.21 (3)	135
N4—H4···O3′′^iv^	0.91	2.12	2.99 (3)	160
N7—H7*A*···O3′′^i^	0.90	2.45	3.24 (3)	147
N7—H7*B*···O4′′	0.90	2.50	3.04 (3)	119

Symmetry codes: (i) *x*, −*y*+1/2, *z*+1/2; (iii) *x*−1, *y*, *z*; (iv) *x*, *y*, *z*+1.
